# Development and validation of a nomogram for identifying prevalent sarcopenia in Chinese patients with Cardiovascular-Kidney-Metabolic Syndrome

**DOI:** 10.3389/fpubh.2026.1887503

**Published:** 2026-07-22

**Authors:** Chengjun Yang, Qiaohui He, Haiwen Liang, Junru Song, Zehuai Wen, Xingying Qiu, Xiankun Chen, Huayang Cai

**Affiliations:** 1 The Second Clinical Medical College, Guangzhou University of Chinese Medicine, Guangzhou, Guangdong, China; 2 Center for Clinical Research, The Second Affiliated Hospital of Guangzhou University of Chinese Medicine, Guangzhou, Guangdong, China; 3 Guangdong Provincial Key Laboratory of Chinese Medicine for Prevention and Treatment of Refractory Chronic Diseases, The Second Affiliated Hospital of Guangzhou University of Chinese Medicine, Guangzhou, China; 4 Department of Scientific Research, Guangdong Provincial Hospital of Chinese Medicine, Guangzhou, Guangdong, China; 5 Guangdong Provincial Key Laboratory of Research on Emergency in TCM, The Second Affiliated Hospital of Guangzhou University of Chinese Medicine, Guangzhou, China

**Keywords:** Cardiovascular-Kidney-Metabolic Syndrome, LASSO, muscle, nomogram, ROC curve, sarcopenia

## Abstract

**Background:**

Sarcopenia is recognized as a significant comorbidity in patients with Cardiovascular-Kidney-Metabolic (CKM) Syndrome, yet validated prediction models for this population remain lacking. This study aimed to develop and validate a nomogram for predicting sarcopenia risk in Chinese patients with CKM syndrome.

**Methods:**

Data were derived from the China Health and Retirement Longitudinal Study (CHARLS) and an independent hospital dataset. The CHARLS 2015 dataset was split into a training set and an internal validation set; the CHARLS 2011 dataset served as the external validation set; and inpatients from Guangdong Provincial Hospital of Chinese Medicine constituted the hospital validation set. Sarcopenia was diagnosed according to the 2025 Asian Working Group for Sarcopenia criteria. Least absolute shrinkage and selection operator (LASSO) regression combined with multivariable logistic regression was used for predictor selection and model development. Model performance was evaluated by discrimination, calibration, and decision curve analysis (DCA).

**Results:**

Nine predictors were identified: age, smoking status, high-density lipoprotein cholesterol, triglycerides, uric acid, C-reactive protein, hemoglobin, chronic obstructive pulmonary disease, and chronic liver disease. The model achieved area under the curve values of 0.817, 0.808, 0.800, and 0.834 in the training, internal validation, external validation, and hospital validation sets, respectively. Calibration was satisfactory in development cohorts (*p* > 0.05), with some calibration drift in external populations. DCA confirmed clinical utility across all datasets.

**Conclusion:**

The developed nomogram incorporating nine accessible predictors demonstrated robust discrimination and clinical applicability for sarcopenia risk assessment in Chinese CKM patients, supporting its use in early screening and individualized intervention.

## Graphical abstract

**Figure:**
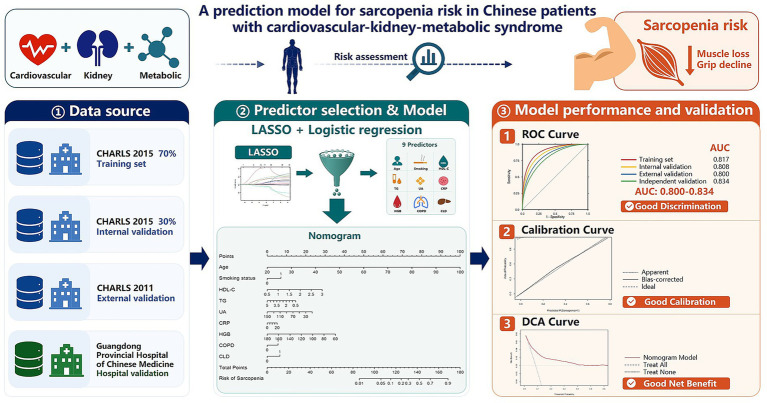

## 1 Introduction

Cardiovascular-Kidney-Metabolic Syndrome (CKM) is identified as a comprehensive disorder that emerges due to the interaction among various metabolic risk elements, including chronic kidney disease (CKD) and cardiovascular disease (CVD) (1). Its core characteristics include mutual promotion of metabolic abnormalities, chronic kidney injury, and cardiovascular disorders, contributing to the dysfunction of multiple organs and higher rate of cardiovascular-related events and mortality. As CKM progresses, it exacerbates the “metabolic-cardiac-renal” vicious cycle, elevating all-cause mortality risk and major adverse cardiovascular event risk (2).

During 2011–2020, the occurrence rate of CKM stages 3 and 4 reached 55.3% among individuals aged ≥65 years in the United States, while 81.8% of adults aged 20–44 years exhibited cardiorenal metabolic risk factors (3). According to nationally representative data, the prevalence of CKM syndrome among Chinese adults aged ≥35 years is extremely widespread, with the weighted prevalence of CKM stages 1–4 exceeding 80% in total, and advanced stages (stages 3–4) accounting for 23.6%. The 5-year follow-up showed that the risks of all-cause mortality, cardiovascular mortality, and non-cardiovascular mortality increased significantly with advancing CKM stages. Compared with stage 0, the risk of all-cause mortality for stage 4 increased to 4.00-fold, and the population attributable mortality burden attributable to higher stages (stages 2–4) was substantial (4).

Despite the increasing recognition of sarcopenia as a clinically significant comorbidity in CKM (5, 6), the development of validated prediction models specifically designed for this population remains limited. Current prediction models predominantly concentrate on isolated disease entities and frequently overlook the complex multifactorial pathophysiology characteristic of CKM syndrome, thereby compromising their clinical applicability and predictive accuracy. Therefore, there is a need to construct a comprehensive and interpretable screening model to identify CKM patients with a high probability of prevalent sarcopenia using routinely accessible clinical variables. This study aimed to develop and validate a nomogram for identifying prevalent sarcopenia among Chinese patients with CKM syndrome.

## 2 Methods

### 2.1 Study design and population

This study was conducted and reported in accordance with the Transparent Reporting of a Multivariable Prediction Model for Individual Prognosis or Diagnosis (TRIPOD) guidelines to ensure transparency and clarity in the development and validation of the prediction model (7). This research used information from two sources: the China Health and Retirement Longitudinal Study (CHARLS) database and a separate clinical dataset from the Guangdong Provincial Hospital of Chinese Medicine.

CHARLS is a nationally representative baseline survey initiated in 2008, covering 450 communities across 28 provinces, with a sample size exceeding 17,000 households (8). The program aims to facilitate interdisciplinary research on aging through the longitudinal tracking of detailed data on personal health, economic status, and social position. The CHARLS study was approved by the Institutional Review Board of Peking University (IRB00001052-11015), and all participants provided written informed consent at the time of enrollment. For this study, CHARLS data from 2011 (wave 1) and 2015 (wave 3) were used, yielding an initial sample of 17,708 and 21,097 participants. The CHARLS 2015 dataset was randomly partitioned into a training set (70%) and an internal validation set (30%), while the CHARLS 2011 dataset served as the external validation set. Participants who were not diagnosed with CKM, individuals with missing key variables information, those with missing or abnormal core variables for CKM diagnosis, and participants with missing or abnormal variables for sarcopenia diagnosis were excluded from the study. More information about CHARLS is available at: http://charls.pku.edu.cn.

**Figure 1:**
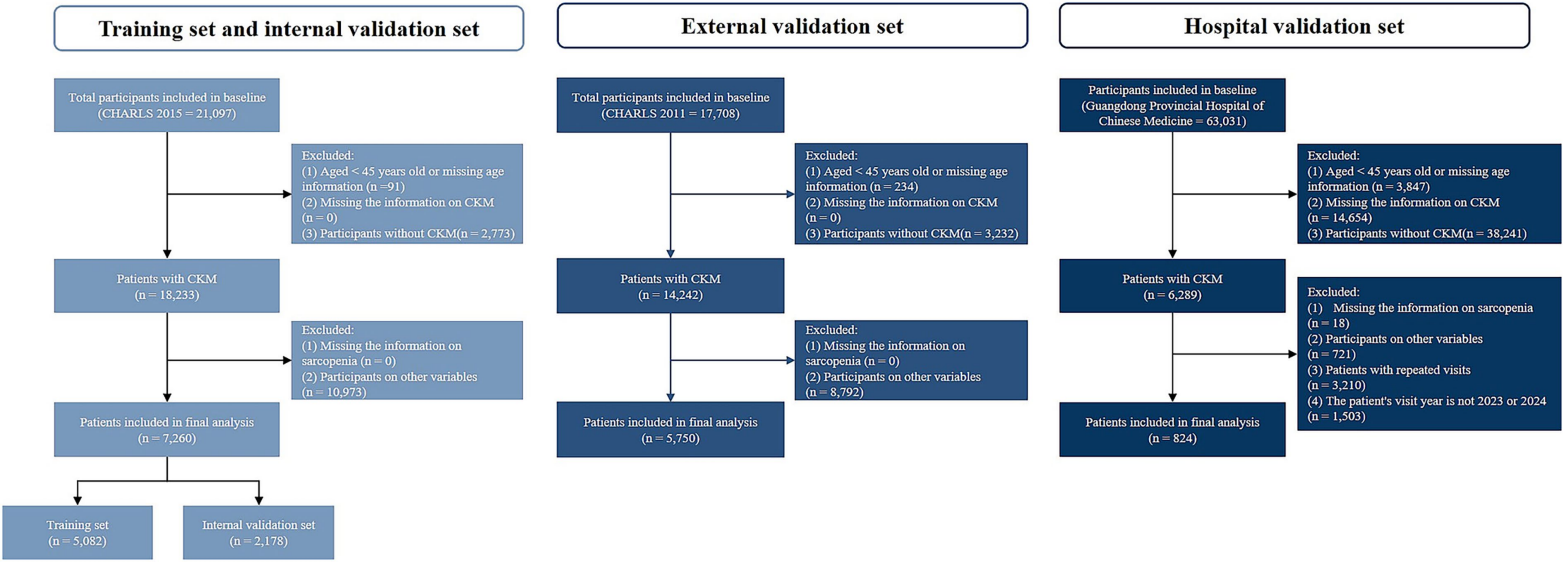
Flowchart illustrating the process of patient selection. CHARLS, China Health and Retirement Longitudinal Study; CKM, Cardiovascular-Kidney-Metabolic Syndrome.

This study preliminarily screened 63,031 samples from hospitalized patients in Guangdong Provincial Hospital of Chinese Medicine during the period from January 1, 2023 to December 31, 2024. To ensure consistency, participants were selected based on the same inclusion criteria set for the CHARLS dataset, encompassing individuals diagnosed with CKM. Ultimately, 824 patients were included in this independent hospital validation set. The hospital validation component of this study was approved by the Ethics Committee of Guangdong Provincial Hospital of Chinese Medicine (ZE2026-113-01). Given that this hospital data analysis is a retrospective observational study, the ethics committee waived the requirement for written informed consent. A detailed flowchart of the inclusion and exclusion process is presented in Figure 1.

### 2.2 CKM syndrome assessment

According to the Presidential Advisory Note issued by the American Heart Association, CKM syndrome was stratified into stages 0–4 (1):

- *Stage 0:* concurrently normal weight, blood pressure, lipid profile, glucose levels, and renal function, with no evidence of clinical or subclinical CVD.
- *Stage 1:* BMI ≥ 23 kg/m^2^, waist circumference ≥90 cm in men or ≥82 cm in women, dysfunctional adipose tissue, but without additional metabolic risk factors or CKD.
- *Stage 2:* presence of metabolic risk factors, hypertension, or diabetes mellitus, or being at moderate-high risk for CKD.
- *Stage 3:* excessive or dysfunctional obesity alongside metabolic risk factors, or subclinical CVD.
- *Stage 4:* established clinical CVD coexisting with excessive/dysfunctional obesity, other metabolic risk factors, or CKD.

For the purposes of this study, participants classified as CKM stages 1 through 4 were defined as the CKM syndrome population. Additionally, participants were excluded based on the following criteria: (1) missing key demographic information; (2) missing or abnormal core variables for CKM diagnosis; (3) missing or abnormal variables for sarcopenia diagnosis; (4) participants not diagnosed with CKM.

### 2.3 Sarcopenia assessment

In this study, sarcopenia was assessed according to the 2025 published diagnostic criteria for sarcopenia (9). The assessment encompassed two core dimensions: muscle strength and appendicular skeletal muscle mass (ASM). The specific criteria are as follows:

- *Muscle strength:* for individuals aged 65 years and older, low muscle strength is defined as grip strength <28 kg in men and <18 kg in women. For those younger than 65 years, low muscle strength is defined as grip strength <34 kg in men and <20 kg in women.
- *Muscle mass:* calculated using a validated formula for the Chinese population (10): ASM = 0.193 × weight (kg) + 0.107 × height (cm) − 4.157 × sex (male = 1, female = 2) − 0.037 × age (years) − 2.631. The cut-off for low muscle mass was set as the sex-specific lowest 20% of SMI among the population (11), with 7.24 kg/m^2^ in males and 5.60 kg/m^2^ in females.

### 2.4 Candidate predictors and variable preprocessing

Variable screening was conducted based on clinical significance and knowledge established in previous studies (11). The candidate predictors were classified into four categories: socio-demographic and anthropometric factors, clinical and laboratory measurements, lifestyle factors, and chronic disease history.

#### 2.4.1 Socio-demographic and anthropometric factors

Socio-demographic and anthropometric factors included age (years), gender (Male/Female), height (cm), and weight (kg). Age and gender were recorded as fundamental demographic variables. Height and weight were measured as basic anthropometric parameters.

#### 2.4.2 Clinical and laboratory measurements

Clinical and laboratory measurements were further subdivided into several physiological domains:

1. Blood pressure indicators: SBP (systolic blood pressure, mmHg) and DBP (diastolic blood pressure, mmHg).
2. Glycemic indicators: FPG (fasting plasma glucose, mmol/L) and HbA1c (hemoglobin A1c, %).
3. Lipid profile: TC (total cholesterol, mmol/L), HDL-C (high-density lipoprotein cholesterol, mmol/L), LDL-C (Low-density lipoprotein cholesterol, mmol/L), and TG (triglycerides, mmol/L).
4. Renal function indicators: eGFR (estimated glomerular filtration rate, mL/min/1.73m^2^), SCr (serum creatinine, μmol/L), and UA (uric acid, μmol/L).
5. Inflammatory and hematological markers: CRP (C-reactive protein, mg/L), WBC (white blood cell count, 10^9^/L), and HGB (hemoglobin, g/L). These markers were selected to reflect the systemic inflammatory state and oxygen-carrying capacity of the participants.

#### 2.4.3 Lifestyle factors

Behavioral factors included smoking status and drinking status. These variables were defined as binary indicators, reflecting whether the participants were current smokers or non-smokers, and whether they currently consumed alcohol or not.

#### 2.4.4 Chronic disease history

Health factors pertaining to medical history were defined based on self-reported diagnoses or established clinical thresholds (8, 9): HTN (hypertension), DM (diabetes mellitus), CKD, COPD (chronic obstructive pulmonary disease), CLD (chronic liver disease), CVD, and arthritis. Excessive obesity was defined as BMI ≥ 25 kg/m^2^.

### 2.5 Statistical analysis

#### 2.5.1 Descriptive statistics

For continuous variables, normally distributed data were expressed as mean ± standard deviation, with between-group comparisons performed using independent samples *t*-tests; non-normally distributed data were expressed as median and interquartile range (IQR), with between-group comparisons analyzed using the rank sum test (Mann–Whitney *U* test). Categorical variables were expressed as frequencies and percentages, and comparisons between groups were analyzed using the *χ*^2^ test or Fisher’s exact test. Extreme outliers in continuous variables were identified as values below Q1 − 1.5 × IQR or above Q3 + 1.5 × IQR and excluded prior to imputation. Maximum missing values for all variables extracted did not exceed 20%, and multiple imputation was used to handle missing data using the mice R package (PMM, *m* = 5, maxit = 50, seed = 123). All analysis variables plus the outcome and CKM status were included in the imputation model. The first imputed dataset was used for subsequent analyses.

#### 2.5.2 Predictor selection

The least absolute shrinkage and selection operator (LASSO) regression analysis was used to select predictors and validate the model. First, training set data were analyzed by LASSO regression to select potential predictors of sarcopenia in people with CKM syndrome. A 10-fold cross-validation was applied to confirm the appropriate tuning parameters (*λ*) for LASSO regression analysis, and the most significant features with non-zero coefficients were screened with the LASSO algorithm. Finally, the selected predictors were included in a multifactor logistic regression analysis. A variance inflation factor (VIF) test was performed to ensure there was no severe multicollinearity, and VIF values for all variables were required to be <4. Variables with *p* values <0.05 in the multifactor logistic regression were included in the final prediction model.

#### 2.5.3 Model development

A prediction model for sarcopenia risk in patients with CKM syndrome was developed based on the final independent predictors, and presented as a comprehensive nomogram by “rms” package in R software.

#### 2.5.4 Model evaluation and validation

The performance of the model was validated in the training set, internal validation set, external validation set, and hospital validation set.

- *Discrimination:* the discriminative ability of the model was evaluated using the area under the receiver operating characteristic (ROC) curve (AUC). Sensitivity and specificity were also calculated.
- *Calibration:* calibration curves were plotted to assess the agreement between the predicted probabilities from the nomogram and the actual observed outcomes. The Hosmer–Lemeshow goodness-of-fit test was used to evaluate calibration, with *p* > 0.05 indicating good fit.
- *Clinical utility:* decision curve analysis (DCA) was performed to evaluate the clinical net benefit and applicability of the nomogram model at various threshold probabilities.

All data analyses were performed using R software (version 4.2.2) and Python (version 3.12). Statistical significance was defined as a two-sided *p* < 0.05.

## 3 Result

### 3.1 Participant characteristics

A total of 13,834 participants diagnosed with CKM syndrome were included in this study. The training set comprised 5,082 participants, with 435 (8.6%) diagnosed with sarcopenia. The internal validation set included 2,178 participants, with 189 (8.7%) having sarcopenia. The external validation set consisted of 5,750 participants, with 382 (6.6%) diagnosed with sarcopenia. Additionally, an independent hospital validation set of 824 inpatients from Guangdong Provincial Hospital of Chinese Medicine was included, with 152 (18.4%) having sarcopenia. Many factors including age, body mass index, hemoglobin, blood pressure, lipid profiles, and comorbidities differed significantly between the two groups (*p* < 0.05). The demographic and clinical characteristics of participants across all four datasets are presented in Table 1, Supplementary Tables S1–4.

**Table 1:** Baseline characteristics of the study population in training set.

Characteristic	Overall	Non-sarcopenia	Sarcopenia	*p*-value
	(*n* = 5,082)	(*n* = 4,647)	(*n* = 435)	
Age (years)	61.00 (53.00, 67.00)	60.00 (53.00, 66.00)	71.00 (63.50, 77.00)	<0.001
Gender				0.009
Male (%)	2,251 (44.3)	2032 (43.7)	219 (50.3)	
Female (%)	2,831 (55.7)	2,615 (56.3)	216 (49.7)	
Height (cm)	157.40 (151.90, 164.10)	157.70 (152.25, 164.30)	153.70 (147.55, 160.00)	<0.001
Weight (kg)	62.40 (55.80, 69.80)	63.50 (57.40, 70.60)	48.60 (44.25, 52.50)	<0.001
SBP (mmHg)	128.50 (116.50, 143.00)	128.50 (116.50, 143.00)	131.00 (117.50, 147.00)	0.004
DBP (mmHg)	76.00 (68.50, 84.00)	76.00 (69.00, 84.00)	73.00 (66.00, 81.25)	<0.001
FPG (mmol/L)	5.41 (5.01, 6.01)	5.41 (5.01, 6.01)	5.31 (4.80, 6.01)	0.017
HbA1c (%)	5.90 (5.60, 6.20)	5.90 (5.60, 6.20)	5.80 (5.50, 6.10)	0.024
TC (mmol/L)	4.74 (4.18, 5.36)	4.76 (4.20, 5.37)	4.58 (4.00, 5.30)	<0.001
HDL-C (mmol/L)	1.26 (1.09, 1.45)	1.26 (1.09, 1.44)	1.33 (1.16, 1.57)	<0.001
LDL-C (mmol/L)	2.64 (2.19, 3.14)	2.66 (2.20, 3.15)	2.53 (2.07, 3.08)	0.003
TG (mmol/L)	1.43 (1.02, 2.08)	1.46 (1.05, 2.12)	1.08 (0.84, 1.55)	<0.001
eGFR (mL/min/1.73 m2)	103.74 (84.97, 124.44)	104.30 (85.67, 124.65)	96.75 (75.93, 120.48)	<0.001
SCr (μmol/L)	67.60 (57.90, 80.10)	67.40 (57.90, 79.60)	69.50 (58.15, 86.15)	0.003
UA (μmol/L)	490.00 (400.00, 590.00)	490.00 (410.00, 590.00)	470.00 (390.00, 580.00)	0.008
CRP (mg/L)	1.60 (0.90, 2.80)	1.60 (0.90, 2.80)	1.50 (0.70, 3.30)	0.268
WBC (10^9/L)	5.80 (4.86, 6.96)	5.84 (4.89, 6.96)	5.60 (4.71, 7.00)	0.078
HGB (g/L)	137.00 (126.00, 149.00)	137.00 (127.00, 149.00)	129.00 (120.00, 141.00)	<0.001
Grip (kg)	28.15 (22.00, 35.75)	29.00 (23.13, 36.50)	17.00 (13.00, 24.37)	<0.001
ASM (kg)	17.11 (14.44, 21.07)	17.38 (14.66, 21.38)	14.01 (11.50, 17.44)	<0.001
Smoking status (%)	2,100 (41.3)	1871 (40.3)	229 (52.6)	<0.001
Alcohol status (%)	2,290 (45.1)	2078 (44.7)	212 (48.7)	0.119
HTN (%)	2,399 (47.2)	2,118 (45.6)	281 (64.6)	<0.001
DM (%)	708 (13.9)	645 (13.9)	63 (14.5)	0.783
CVD (%)	1,357 (26.7)	1,171 (25.2)	186 (42.8)	<0.001
CKD (%)	540 (10.6)	475 (10.2)	65 (14.9)	0.003
COPD (%)	693 (13.6)	581 (12.5)	112 (25.7)	<0.001
CLD (%)	348 (6.8)	305 (6.6)	43 (9.9)	0.012
Arthritis (%)	2,279 (44.8)	2034 (43.8)	245 (56.3)	<0.001

SBP, systolic blood pressure; DBP, diastolic blood pressure; FPG, fasting plasma glucose; HbA1c, hemoglobin A1c; TC, total cholesterol; HDL-C, high-density lipoprotein cholesterol; LDL-C, low-density lipoprotein cholesterol; TG, triglycerides; eGFR, estimated glomerular filtration rate; SCr, serum creatinine; UA, uric acid; CRP, C-reactive protein; WBC, white blood cell count; HGB, hemoglobin; ASM, appendicular skeletal muscle mass; HTN, hypertension; DM, diabetes mellitus; CVD, cardiovascular disease; CKD, chronic kidney disease; COPD, chronic obstructive pulmonary disease; CLD, chronic liver disease.

### 3.2 Predictor selection

**Figure 2:**
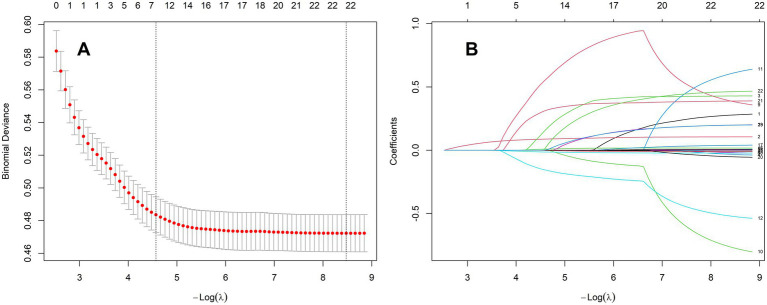
Demographic and clinical feature selection using the LASSO regression model. **(A)** According to the logarithmic (lambda) sequence, a coefficient profile was generated, and non-zero coefficients were produced by the optimal lambda. **(B)** The optimal parameter (lambda) in the LASSO model was selected via tenfold cross-validation using minimum criteria. The partial likelihood deviation (binomial deviation) curve relative to log (lambda) was plotted. A virtual vertical line at the optimal value was drawn using one SE of minimum criterion (the 1-SE criterion).

Of the 22 candidate predictors, LASSO regression with 10-fold cross-validation identified 9 variables with non-zero coefficients at the optimal tuning parameter (lambda.1se = 0.01037): age, smoking status, HDL-C, TG, UA, CRP, HGB, COPD, CLD (Figure 2). These 9 variables were entered into a multivariable logistic regression analysis. The results demonstrated that all 9 variables remained statistically significant (*p* < 0.05) in the multivariable model (Table 2). The variance inflation factor (VIF) for all predictors ranged from 1.014 to 1.270, confirming the absence of severe multicollinearity (VIF < 4). Among the significant predictors, age, current smoking, HDL-C, CRP, COPD, and CLD were positively associated with the risk of sarcopenia in the CKM population, while TG, UA, and HGB were negatively associated with the risk of sarcopenia in the CKM population.

**Table 2:** Multivariable logistic regression analysis of sarcopenia in patients with CKM syndrome.

Variables	*β*	OR	95% CI	*p*-value
Intercept	−6.437	–	–	–
Age	0.103	1.108	(1.095, 1.122)	<0.001
Smoking status	0.581	1.789	(1.404, 2.278)	<0.001
HDL-C	0.936	2.55	(1.773, 3.667)	<0.001
TG	−0.269	0.764	(0.658, 0.888)	<0.001
UA (per 100 μmol/L)	−0.014	0.986	(0.979, 0.994)	0.001
CRP	0.021	1.021	(1.010, 1.033)	<0.001
HGB	−0.024	0.976	(0.970, 0.983)	<0.001
COPD	0.456	1.578	(1.210, 2.057)	0.001
CLD	0.539	1.714	(1.184, 2.482)	0.004

*β*, regression coefficient; OR, odds ratio; CI, confidence interval; HDL-C, high-density lipoprotein cholesterol; TG, triglycerides; UA, uric acid; CRP, C-reactive protein; HGB, hemoglobin; COPD, chronic obstructive pulmonary disease; CLD, chronic liver disease.

### 3.3 Model development and nomogram

**Figure 3:**
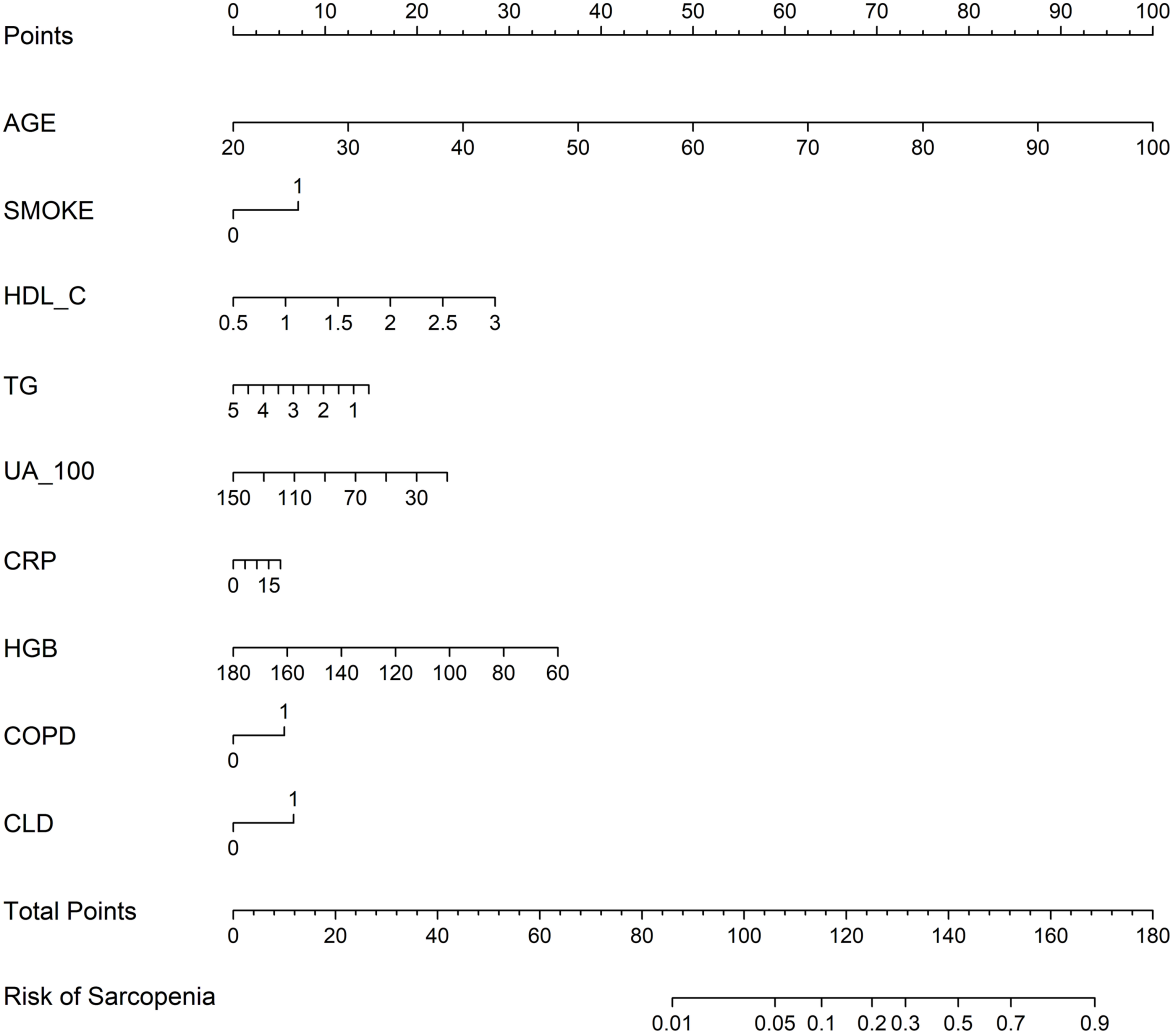
Nomogram to evaluate the risk of sarcopenia in patients with CKM. HDL-C, high-density lipoprotein cholesterol; TG, triglycerides; UA, uric acid; CRP, C-reactive protein; HGB, hemoglobin; COPD, chronic obstructive pulmonary disease; CLD, chronic liver disease.

A multivariable logistic regression-based nomogram was constructed to predict the risk of sarcopenia in patients with CKM syndrome, incorporating all 9 independent predictors identified from the LASSO and multivariable regression pipeline (Figure 3). The nomogram assigns a weighted score to each predictor based on its regression coefficient, which is summed to generate a total score that can be directly translated into an individualized predicted probability of sarcopenia.

### 3.4 Model performance and validation

#### 3.4.1 Discrimination

**Figure 4:**
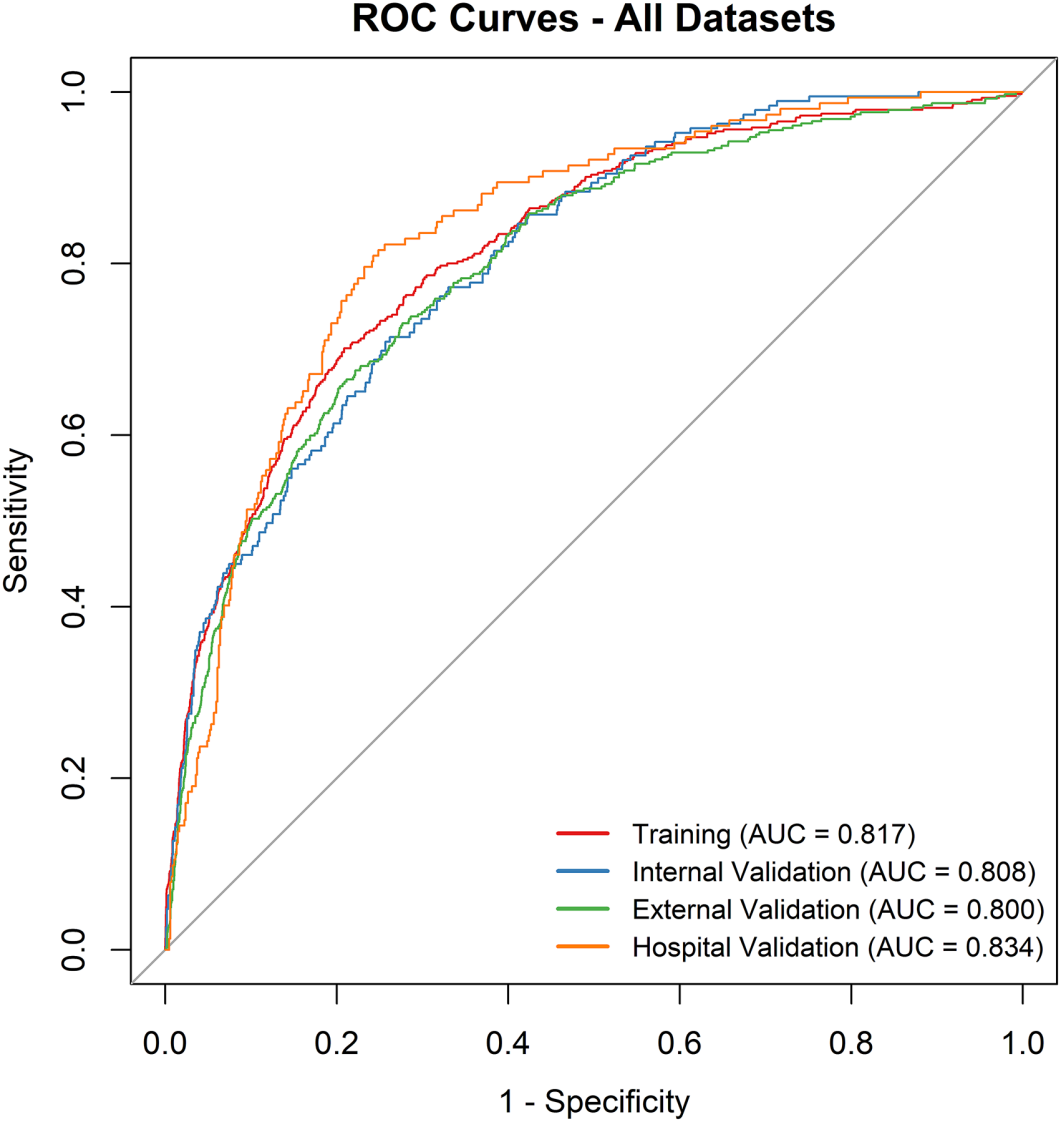
ROC curve.

The area under the receiver operating characteristic curve (AUC) was employed to evaluate the discriminative ability of the nomogram across four datasets. The model demonstrated satisfactory discrimination in the training set (AUC = 0.817, 95% CI: 0.796–0.838), internal validation set (AUC = 0.808, 95% CI: 0.778–0.838), external validation set (AUC = 0.800, 95% CI: 0.777–0.823), and hospital validation set (AUC = 0.834, 95% CI: 0.801–0.868) (Table 3, Figure 4). At the optimal threshold probability of 0.103 in the training set, the model achieved a sensitivity of 0.701 and specificity of 0.791. The hospital validation set demonstrated the highest performance, with a sensitivity of 0.816 and specificity of 0.751 at a threshold of 0.257, reflecting robust generalizability to real-world clinical populations.

**Table 3:** Performance of the nomogram across datasets.

Dataset	AUC (95% CI)	Sensitivity	Specificity	HL *p*-value
Training	0.817 (0.796–0.838)	0.701	0.791	0.1280
Internal validation	0.808 (0.778–0.838)	0.714	0.738	0.0705
External validation	0.800 (0.777–0.823)	0.730	0.723	0.0212
Hospital validation	0.834 (0.801–0.868)	0.816	0.751	<0.001

AUC, area under the receiver operating characteristic curve; CI, confidence interval; HL, Hosmer–Lemeshow test.

#### 3.4.2 Calibration

**Figure 5:**
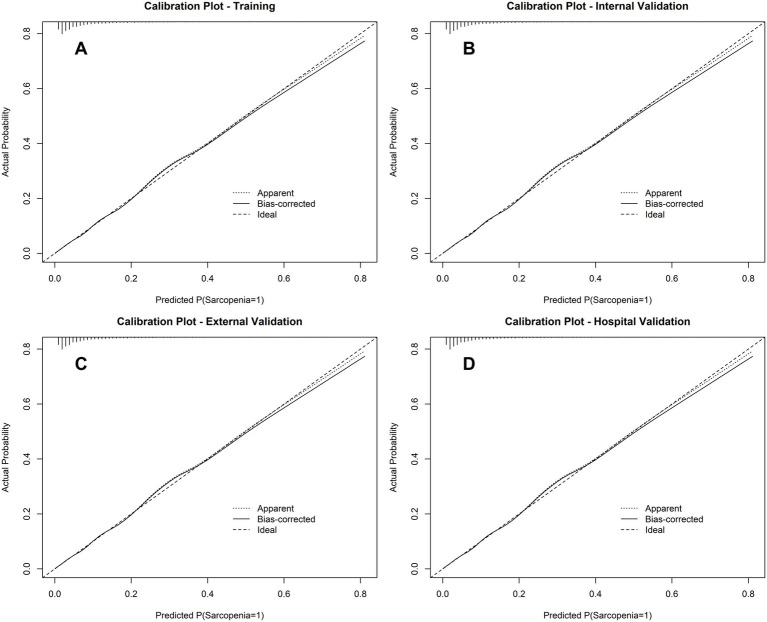
Calibration curves. **(A)** Calibration curves for training set. **(B)** Calibration curves for internal validation set. **(C)** Calibration curves for external validation set. **(D)** Calibration curves for hospital validation set.

The nomogram was evaluated using calibration plots (Figure 5) and the Hosmer–Lemeshow goodness-of-fit test. The Hosmer–Lemeshow test showed satisfactory calibration in the training set (*χ*^2^ = 12.559, *p* = 0.128) and internal validation set (*χ*^2^ = 14.462, *p* = 0.071). However, the test indicated suboptimal calibration in the external validation set (*p* = 0.021) and hospital validation set (*p* = 0.001), suggesting calibration drift when applied to different populations. This may reflect differences in disease severity, measurement methods, and population characteristics between community-based and hospital-based cohorts.

#### 3.4.3 Clinical utility

**Figure 6:**
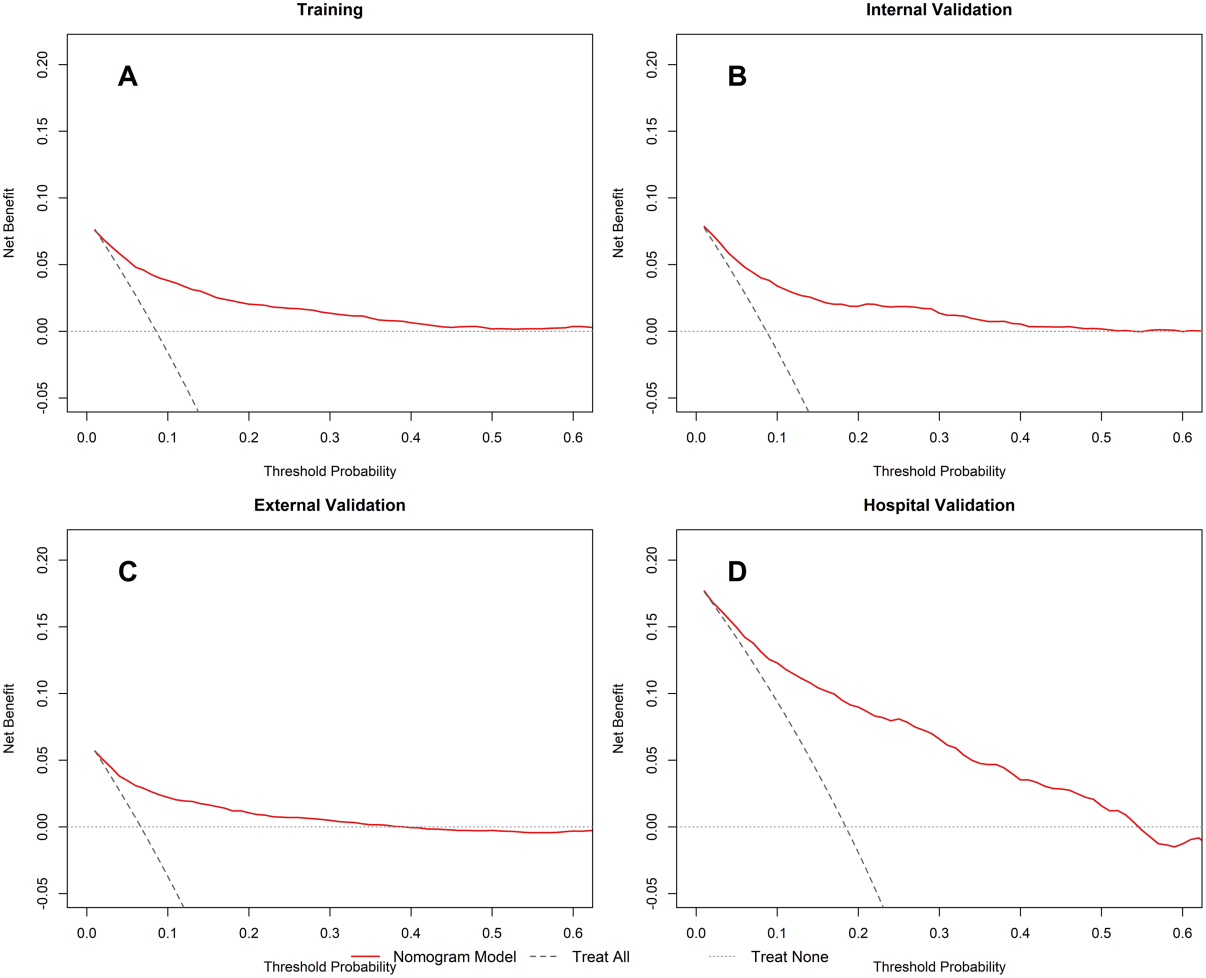
DCA curves. **(A)** DCA curves for training set. **(B)** DCA curves for internal validation set. **(C)** DCA curves for external validation set. **(D)** DCA curves for hospital validation set.

DCA was performed to evaluate the net clinical benefit of the nomogram across a range of threshold probabilities. In all four datasets, the nomogram model consistently demonstrated a higher net benefit compared with the “treat all” and “treat none” strategies over clinically relevant threshold probability ranges (Figures 6A–D). The DCA results indicate that the nomogram-guided decision strategy yields meaningful clinical utility for sarcopenia risk screening in CKM syndrome patients.

## 4 Discussion

Both CKM and sarcopenia are common age-related disorders, and their coexistence significantly increases the risk of adverse outcomes in patients, including elevated all-cause mortality and cardiovascular event rates (5). Although sarcopenia is increasingly recognized as an important clinical comorbidity in patients with CKM, sarcopenia diagnostic prediction models specifically designed for this population remain limited. This study, based on data from CHARLS and an independent hospital dataset, developed and validated a nomogram model incorporating nine easily accessible predictors (age, smoking status, HDL-C, TG, UA, CRP, HGB, COPD, and CLD). The nomogram demonstrated robust discrimination across all validation cohorts (AUC 0.800–0.834), indicating strong ability to rank individuals by sarcopenia risk. While calibration was satisfactory in the development cohorts, the significant Hosmer–Lemeshow tests in external populations highlight important considerations for model transportability. The calibration drift observed in the hospital validation set (*p* = 0.001) likely reflects fundamental differences between community-dwelling and hospitalized CKM patients, including higher disease severity (sarcopenia prevalence 18.4% vs. 6.6–8.7%), different comorbidity profiles, and potential measurement heterogeneity. Such calibration challenges are commonly observed when prediction models are applied across different healthcare settings and emphasize the need for local validation and potential recalibration before clinical implementation in specific populations. Recent studies have highlighted the translational significance of biomarker-based and interpretable clinical modeling, such as utilizing proteomics for cardiovascular biomarker discovery (12) and developing interpretable quantification systems for atherosclerotic risk (13). Aligning with these conceptual frameworks, our nomogram integrates routinely accessible systemic biomarkers to provide a transparent, pragmatic tool for personalized risk stratification in CKM patients.

This study found that aging is associated with the risk of sarcopenia in CKM. Epidemiological studies have confirmed that the global prevalence of sarcopenia among older adults rises sharply with age, and is particularly pronounced in those aged 80 and above (14). Data from a large-scale national survey in South Korea further confirmed this age-related epidemiological pattern: the prevalence of sarcopenia among older adults aged 60 and above was 5.5–7.9%, while among those aged 80 and above, it rose to 21.5–25.9% (15). A systematic review and meta-analysis reported that the prevalence of sarcopenia was 15.7% among adults aged 60–69 years, increased to 27.2% in those aged 70–79 years, and reached 45.4% in individuals aged 80 years and older (16). The pathophysiological mechanisms underlying age-related sarcopenia are multifactorial and complex. Age-related sarcopenia is driven by mitochondrial dysfunction and chronic low-grade inflammation, which promote oxidative stress and muscle protein degradation (17, 18).

The results of this study showed that current smokers have a higher risk of sarcopenia than non-smokers. A large cross-sectional study involving 783 sarcopenia cases and 4,918 non-sarcopenic older adults in Zhejiang, China, reported that current smokers had a 78.6% higher risk of sarcopenia compared with never-smokers (OR = 1.786, 95% CI: 1.387–2.301), with a significant linear dose–response relationship between cumulative pack-years and sarcopenia risk (19). Furthermore, a systematic review and meta-analysis of 45 studies encompassing 37,571 older adults confirmed smoking as an independent risk factor for sarcopenia in this population (16). Mechanistically, smoking impairs muscle protein synthesis, upregulates myostatin and MAFbx, and induces systemic oxidative stress and inflammation, collectively disrupting the balance between muscle protein synthesis and degradation (20, 21).

In this study, high HDL-C was positively correlated with the risk of sarcopenia. HDL-C is often regarded as good cholesterol (22), but multiple epidemiological studies have reached contradictory conclusions, finding that it is associated with an increased risk of sarcopenia and low muscle mass. Longitudinal studies from the CHARLS database show that elevated HDL-C was associated with the risk of incident sarcopenia and low grip strength (22). At the level of evidence-based medicine, a meta-analysis integrating 20 studies showed that the sarcopenic population had significantly higher HDL-C levels than the non-sarcopenic population (23). Mendelian randomization analyses further suggested a bidirectional causal relationship between genetically predicted HDL-C and appendicular lean mass, supporting a potential causal link (24). The underlying mechanisms may involve aging-related alterations in HDL-C metabolism and a pro-inflammatory shift under chronic conditions (25–27).

This study found that low TG levels were associated with the occurrence of sarcopenia. A longitudinal evidence from a 4-year prospective study of 224 community-dwelling older women further showed that an increase in TG levels over the follow-up period was associated with a reduced likelihood of possible sarcopenia, suggesting that preserving or increasing TG levels may protect against muscle loss in older adults (28). A study based on the national CHARLS cohort found that a higher TG/HDL-C ratio is inversely associated with the risk of incident sarcopenia. This indicates that, assuming HDL-C remains constant, elevated TG levels correspond to a lower risk of sarcopenia; conversely, assuming TG remains constant, elevated HDL-C levels correspond to a higher risk. These findings are consistent with our earlier conclusions (29). Perna et al. (30) showed that increasing obesity and metabolic parameters within the reference range may help prevent muscle loss. Jiang et al. (31) also demonstrated that the prevalence of sarcopenia decreases with increasing body fat percentage. However, elevated TG are themselves an important component and risk factor of CKM (1). In this clinical context, the implications of these findings must be interpreted with particular caution. Therefore, the potential protective effect of elevated TG levels against sarcopenia presents a clinical paradox: intensive lipid-lowering strategies aimed at reducing cardiovascular risk may inadvertently increase the risk of sarcopenia, whereas allowing TG levels to remain elevated to preserve muscle mass may exacerbate adverse cardiovascular and metabolic outcomes. Hence, future research should explore the optimal TG range for this specific population, striking a balance between CKM protection and sarcopenia prevention.

This study found that lower UA levels were associated with the risk of sarcopenia. A cross-sectional study by Baker et al. (32) found that low serum UA concentration was associated with low muscle mass and with increased mortality. A longitudinal analysis further confirmed that, among the male population, higher UA levels were significantly associated with a lower risk of incident sarcopenia (33). These findings were corroborated by the InCHIANTI study, which prospectively demonstrated that higher circulating UA levels were independently associated with better handgrip strength and knee extension torque in older persons over 3 years of follow-up (34). The protective effect of UA may be attributed to its antioxidant capacity in mitigating oxidative stress-induced muscle damage (33, 35).

This study found that CRP, as a predictor in the model, was positively correlated with the risk of sarcopenia. A meta-analysis including over 89,000 participants reported that higher CRP levels were significantly negatively associated with skeletal muscle strength and mass (36), while low skeletal muscle strength and mass are direct diagnostic criteria for sarcopenia. A study by Lin et al. (37) that combined cross-sectional analysis with Mendelian randomization also reached the same conclusion. CRP is a downstream marker of pro-inflammatory cytokines that promote muscle protein degradation and suppress anabolic signaling (36, 38). Converging lines of evidence from multiple studies indicate that high levels of C-reactive protein promote muscle catabolism and are a risk factor for sarcopenia.

This study found that HGB levels were negatively correlated with sarcopenia. A meta-analysis by Wang and Lin (39) showed that hemoglobin levels in patients with sarcopenia were significantly lower than those in the control group, indicating a robust negative association with sarcopenia across different populations. Liu et al. (40) showed that each 1 g/dL increase in HGB level was associated with a 5% reduction in the odds of sarcopenia, and that HGB was negatively correlated with low appendicular skeletal muscle mass. Low HGB may contribute to sarcopenia through tissue hypoxia, impaired nutritional status, and reduced physical activity (41–43).

The results of this study showed that COPD is closely associated with sarcopenia. A meta-analysis by Yu et al. (44) showed that the prevalence of sarcopenia in the COPD population was 29%, and individuals with COPD had a 1.51-fold higher risk of sarcopenia compared to those without COPD. COPD promotes sarcopenia through systemic inflammation, oxidative stress, and anabolic-catabolic imbalance, which collectively accelerate muscle protein degradation (45–49).

The results of this study showed that CLD is one of the predictors of sarcopenia in CKM, and the occurrence of CLD is associated with a higher risk of sarcopenia. Clinical research has established sarcopenia as an independent prognostic factor across the CLD spectrum. Cirrhotic patients with sarcopenia have a 2.13 fold increased risk of mortality and a 2.27 fold increased risk of hepatic encephalopathy (50). In liver transplant candidates, pre-transplant sarcopenia is significantly associated with prolonged ICU stay, increased postoperative complications, and higher 1-year mortality (51, 52). The pathophysiology is driven by hyperammonemia, which activates the NF-κB-myostatin axis, suppressing protein synthesis and promoting autophagy-mediated proteolysis (53, 54). Additional mechanisms, including chronic inflammation, insulin resistance, and malnutrition, synergistically exacerbate muscle wasting (55).

The inclusion of classical metabolic and cardiovascular indicators, including TG, HDL-C, and UA, as key predictors in our model carries meaningful implications for both clinical interpretation and disease management. Traditionally, these markers are interpreted primarily in the context of CKM progression rather than as specific signals of sarcopenia risk. Their prominent role in our nomogram reinforces the concept of metabolic sarcopenia, indicating that skeletal muscle decline in the CKM population arises largely from systemic metabolic disturbances, such as lipotoxicity and oxidative stress. In clinical practice, this finding suggests that sarcopenia risk assessment can be readily incorporated into routine metabolic monitoring.

### 4.1 Limitations

There are some limitations in this study. First, the potential predictors included in this study were variables common to both the CHARLS database and hospital medical records, which introduces limitations in variable selection. Second, the predictive model was constructed and validated based on Chinese data, and further validation using data from other regions is needed. Third, this study is a cross-sectional study and cannot establish causal relationships between the predictors and outcomes (11). Fourth, while the model showed good discrimination, calibration performance varied across validation cohorts. The significant Hosmer–Lemeshow test in external and hospital validation sets suggests the model may require recalibration when applied to populations with substantially different characteristics. The CHARLS and hospital cohorts differed in participant characteristics, disease severity, sarcopenia prevalence, and clinical settings, which may have contributed to the observed calibration drift. Future studies should explore calibration strategies, such as model updating or local recalibration, to improve transportability across diverse clinical contexts. Additionally, sarcopenia diagnosis in this study relied on an anthropometric equation rather than direct measurements such as DXA or BIA, which is a limitation inherent to large-scale epidemiological studies. Nevertheless, this equation has been validated against BIA in the CHARLS population with excellent agreement (ICC = 0.933, 56). Finally, owing to the limitations of the available variables in our datasets, a head-to-head comparison between our nomogram and existing screening tools (e.g., SARC-F or the Ishii score) could not be performed. Although integrating systemic metabolic biomarkers theoretically provides specific clinical insights for the CKM population, the exact incremental value of our model requires empirical evaluation. Future prospective observational studies are planned to compare our tool with conventional scores and further validate its clinical utility.

## 5 Conclusion

This study developed and validated a nomogram model for predicting the risk of sarcopenia in patients with CKM. Our nomogram incorporated age, smoking status, HDL-C, TG, UA, CRP, HGB, COPD, and CLD, and was proven to be an effective tool for risk assessment in the internal validation, external validation, and hospital validation cohorts. The developed prediction model holds significant value for screening the risk of sarcopenia in patients with CKM.

## Data Availability

The CHARLS data was sourced from the CHARLS public database, and the original data can be obtained at the website http://charls.pku.edu.cn. Due to ethical limitations, data from Guangdong Provincial Hospital of Chinese Medicine is not publicly available unless the researcher has legitimate reasons for requesting access and obtains the consent of the corresponding author.
