# Supplementary material for: Development and validation of a nomogram for identifying prevalent sarcopenia in Chinese patients with Cardiovascular-Kidney-Metabolic Syndrome
Source: Front Public Health. 2026 Jul 22;14:1887503. doi: 10.3389/fpubh.2026.1887503 (PMC13437309; doi:10.3389/fpubh.2026.1887503)
Supplement: Supplementary file 1 [file Data_Sheet_1.DOCX]

Supplementary Material

# 1 Supplementary Tables

## 1.1 Table S1 Baseline characteristics of the study population in internal validation set

| **Characteristic** | **Overall** | **Non-sarcopenia** | **Sarcopenia** | **p-value** |
| --- | --- | --- | --- | --- |
|  | **(n=2178)** | **(n=1989)** | **(n=189)** |  |
| **Age (years)** | 61.00 (54.00, 68.00) | 60.00 (53.00, 67.00) | 70.00 (63.00, 78.00) | <0.001 |
| **Gender** |  |  |  | 0.473 |
| Male (%) | 943 (43.3) | 856 (43.0) | 87 (46.0) |  |
| Female (%) | 1235 (56.7) | 1133 (57.0) | 102 (54.0) |  |
| **Height (cm)** | 157.50 (151.60, 163.77) | 157.80 (152.10, 164.10) | 152.80 (147.60, 159.70) | <0.001 |
| **Weight (kg)** | 62.50 (55.40, 69.90) | 63.70 (57.00, 70.60) | 48.70 (44.10, 52.50) | <0.001 |
| **SBP (mmHg)** | 129.50 (116.00, 144.50) | 129.00 (115.50, 144.00) | 134.50 (118.00, 149.50) | 0.004 |
| **DBP (mmHg)** | 76.00 (68.50, 84.50) | 76.00 (69.00, 84.50) | 74.50 (66.50, 84.50) | 0.052 |
| **FPG (mmol/L)** | 5.41 (5.01, 6.01) | 5.41 (5.01, 6.01) | 5.31 (4.80, 5.81) | 0.016 |
| **HbA1c (%)** | 5.80 (5.50, 6.20) | 5.80 (5.50, 6.20) | 5.80 (5.50, 6.10) | 0.229 |
| **TC (mmol/L)** | 4.76 (4.21, 5.41) | 4.76 (4.22, 5.41) | 4.72 (4.06, 5.44) | 0.545 |
| **HDL-C (mmol/L)** | 1.26 (1.10, 1.47) | 1.26 (1.10, 1.45) | 1.41 (1.13, 1.66) | <0.001 |
| **LDL-C (mmol/L)** | 2.66 (2.16, 3.16) | 2.66 (2.17, 3.16) | 2.68 (2.09, 3.12) | 0.517 |
| **TG (mmol/L)** | 1.44 (1.02, 2.08) | 1.48 (1.04, 2.14) | 1.11 (0.86, 1.57) | <0.001 |
| **eGFR (mL/min/1.73m2)** | 104.91 (85.66, 126.22) | 105.49 (86.39, 126.61) | 101.88 (76.77, 119.75) | 0.009 |
| **SCr (umol/L)** | 66.80 (57.40, 79.50) | 66.60 (57.30, 79.10) | 67.50 (58.70, 86.20) | 0.147 |
| **UA (umol/L)** | 480.00 (400.00, 580.00) | 490.00 (400.00, 580.00) | 480.00 (380.00, 600.00) | 0.211 |
| **CRP (mg/L)** | 1.60 (0.90, 2.80) | 1.60 (0.90, 2.80) | 1.30 (0.70, 2.70) | 0.032 |
| **WBC (10^9/L)** | 5.80 (4.90, 6.90) | 5.84 (4.90, 6.90) | 5.60 (4.70, 6.90) | 0.161 |
| **HGB (g/L)** | 136.00 (126.00, 148.00) | 137.00 (127.00, 149.00) | 131.00 (122.00, 141.00) | <0.001 |
| **Grip (kg)** | 28.25 (22.25, 35.50) | 29.25 (23.30, 36.25) | 17.30 (13.80, 23.00) | <0.001 |
| **ASM (kg)** | 17.14 (14.30, 21.05) | 17.35 (14.62, 21.34) | 13.14 (11.18, 17.36) | <0.001 |
| **Smoking status (%)** | 880 (40.4) | 789 (39.7) | 91 (48.1) | 0.028 |
| **Alcohol status (%)** | 951 (43.7) | 871 (43.8) | 80 (42.3) | 0.756 |
| **HTN (%)** | 1013 (46.5) | 893 (44.9) | 120 (63.5) | <0.001 |
| **DM (%)** | 308 (14.1) | 277 (13.9) | 31 (16.4) | 0.410 |
| **CVD (%)** | 578 (26.5) | 499 (25.1) | 79 (41.8) | <0.001 |
| **CKD (%)** | 214 (9.8) | 191 (9.6) | 23 (12.2) | 0.315 |
| **COPD (%)** | 329 (15.1) | 280 (14.1) | 49 (25.9) | <0.001 |
| **CLD (%)** | 147 (6.7) | 134 (6.7) | 13 (6.9) | 1.000 |
| **Arthritis (%)** | 957 (43.9) | 859 (43.2) | 98 (51.9) | 0.027 |

SBP, systolic blood pressure; DBP, diastolic blood pressure; FPG, fasting plasma glucose; HbA1c, hemoglobin A1c; TC, total cholesterol; HDL-C, high-density lipoprotein cholesterol; LDL-C, low-density lipoprotein cholesterol; TG, triglycerides; eGFR, estimated glomerular filtration rate; SCr, serum creatinine; UA, uric acid; CRP, C-reactive protein; WBC, white blood cell count; HGB, hemoglobin; ASM, appendicular skeletal muscle mass; HTN, hypertension; DM, diabetes mellitus; CVD, cardiovascular disease; CKD, chronic kidney disease; COPD, chronic obstructive pulmonary disease; CLD, chronic liver disease.

## 1.2 Table S2 Baseline characteristics of the study population in external validation set

| **Characteristic** | **Overall** | **Non-sarcopenia** | **Sarcopenia** | **p-value** |
| --- | --- | --- | --- | --- |
|  | **(n=5750)** | **(n=5368)** | **(n=382)** |  |
| **Age (years)** | 58.00 (51.00, 65.00) | 58.00 (51.00, 64.00) | 68.50 (61.25, 76.00) | <0.001 |
| **Gender** |  |  |  | 0.002 |
| Male (%) | 2465 (42.9) | 2272 (42.3) | 193 (50.5) |  |
| Female (%) | 3285 (57.1) | 3096 (57.7) | 189 (49.5) |  |
| **Height (cm)** | 157.50 (152.00, 164.00) | 157.90 (152.30, 164.20) | 152.95 (147.00, 159.80) | <0.001 |
| **Weight (kg)** | 62.50 (55.90, 69.70) | 63.35 (57.20, 70.30) | 48.90 (44.50, 52.80) | <0.001 |
| **SBP (mmHg)** | 130.00 (117.00, 146.00) | 130.00 (117.00, 145.00) | 138.25 (117.12, 155.25) | <0.001 |
| **DBP (mmHg)** | 76.50 (69.00, 85.50) | 77.00 (69.00, 85.50) | 75.50 (66.00, 84.50) | 0.004 |
| **FPG (mmol/L)** | 5.78 (5.32, 6.46) | 5.78 (5.33, 6.46) | 5.68 (5.28, 6.39) | 0.097 |
| **HbA1c (%)** | 5.20 (4.90, 5.50) | 5.20 (4.90, 5.50) | 5.20 (4.90, 5.50) | 0.870 |
| **TC (mmol/L)** | 4.98 (4.39, 5.66) | 4.99 (4.40, 5.67) | 4.90 (4.29, 5.50) | 0.028 |
| **HDL-C (mmol/L)** | 1.21 (1.00, 1.46) | 1.20 (0.99, 1.44) | 1.39 (1.13, 1.63) | <0.001 |
| **LDL-C (mmol/L)** | 3.02 (2.47, 3.64) | 3.03 (2.48, 3.65) | 2.88 (2.34, 3.46) | 0.008 |
| **TG (mmol/L)** | 1.32 (0.93, 1.93) | 1.34 (0.94, 1.96) | 1.08 (0.77, 1.57) | <0.001 |
| **eGFR (mL/min/1.73m2)** | 105.94 (87.25, 126.61) | 106.74 (87.79, 127.06) | 98.93 (77.95, 118.30) | <0.001 |
| **SCr (umol/L)** | 66.93 (57.94, 78.91) | 66.93 (57.94, 78.91) | 68.93 (59.19, 82.91) | <0.001 |
| **UA (umol/L)** | 436.80 (362.42, 523.11) | 435.96 (362.21, 522.14) | 451.16 (367.29, 542.43) | 0.075 |
| **CRP (mg/L)** | 1.18 (0.63, 2.34) | 1.18 (0.64, 2.33) | 1.11 (0.60, 2.68) | 0.700 |
| **WBC (10^9/L)** | 6.02 (5.00, 7.30) | 6.05 (5.00, 7.30) | 6.00 (5.00, 7.17) | 0.422 |
| **HGB (g/L)** | 143.00 (131.00, 156.00) | 144.00 (132.00, 157.00) | 138.00 (127.00, 149.00) | <0.001 |
| **Grip (kg)** | 30.00 (23.50, 37.95) | 30.50 (24.50, 38.50) | 17.50 (14.06, 24.49) | <0.001 |
| **ASM (kg)** | 17.20 (14.52, 21.07) | 17.39 (14.71, 21.28) | 14.33 (11.32, 17.43) | <0.001 |
| **Smoking status (%)** | 2040 (35.5) | 1860 (34.6) | 180 (47.1) | <0.001 |
| **Alcohol status (%)** | 2117 (36.8) | 1962 (36.5) | 155 (40.6) | 0.128 |
| **HTN (%)** | 2338 (40.7) | 2100 (39.1) | 238 (62.3) | <0.001 |
| **DM (%)** | 541 (9.4) | 489 (9.1) | 52 (13.6) | 0.005 |
| **CVD (%)** | 1182 (20.6) | 1042 (19.4) | 140 (36.6) | <0.001 |
| **CKD (%)** | 361 (6.3) | 330 (6.1) | 31 (8.1) | 0.155 |
| **COPD (%)** | 531 (9.2) | 456 (8.5) | 75 (19.6) | <0.001 |
| **CLD (%)** | 212 (3.7) | 191 (3.6) | 21 (5.5) | 0.071 |
| **Arthritis (%)** | 2084 (36.2) | 1923 (35.8) | 161 (42.1) | 0.015 |

SBP, systolic blood pressure; DBP, diastolic blood pressure; FPG, fasting plasma glucose; HbA1c, hemoglobin A1c; TC, total cholesterol; HDL-C, high-density lipoprotein cholesterol; LDL-C, low-density lipoprotein cholesterol; TG, triglycerides; eGFR, estimated glomerular filtration rate; SCr, serum creatinine; UA, uric acid; CRP, C-reactive protein; WBC, white blood cell count; HGB, hemoglobin; ASM, appendicular skeletal muscle mass; HTN, hypertension; DM, diabetes mellitus; CVD, cardiovascular disease; CKD, chronic kidney disease; COPD, chronic obstructive pulmonary disease; CLD, chronic liver disease.

## 1.3 Table S3 Baseline characteristics of the study population in hospital validation set

| **Characteristic** | **Overall** | **Non-sarcopenia** | **Sarcopenia** | **p-value** |
| --- | --- | --- | --- | --- |
|  | **(n=824)** | **(n=672)** | **(n=152)** |  |
| **Age (years)** | 70.00 (62.00, 78.00) | 68.00 (61.00, 75.00) | 80.00 (74.75, 86.00) | <0.001 |
| **Gender** |  |  |  | 0.016 |
| Male (%) | 406 (49.3) | 345 (51.3) | 61 (40.1) |  |
| Female (%) | 418 (50.7) | 327 (48.7) | 91 (59.9) |  |
| **Height (cm)** | 161.00 (156.00, 169.00) | 162.50 (157.00, 170.00) | 157.00 (152.00, 165.00) | <0.001 |
| **Weight (kg)** | 66.00 (59.00, 74.43) | 69.00 (62.70, 76.00) | 54.00 (50.00, 57.00) | <0.001 |
| **SBP (mmHg)** | 135.00 (123.00, 147.00) | 135.00 (123.00, 147.00) | 135.00 (122.00, 146.00) | 0.469 |
| **DBP (mmHg)** | 76.00 (67.00, 85.00) | 77.00 (68.00, 86.00) | 70.00 (61.00, 77.25) | <0.001 |
| **FPG (mmol/L)** | 6.97 (5.73, 8.91) | 6.96 (5.71, 9.02) | 7.03 (5.99, 8.52) | 0.567 |
| **HbA1c (%)** | 6.10 (5.70, 6.80) | 6.10 (5.80, 6.83) | 6.10 (5.70, 6.80) | 0.448 |
| **TC (mmol/L)** | 4.37 (3.58, 5.28) | 4.39 (3.65, 5.29) | 4.25 (3.29, 5.19) | 0.052 |
| **HDL-C (mmol/L)** | 1.15 (0.96, 1.35) | 1.14 (0.95, 1.34) | 1.15 (0.98, 1.38) | 0.326 |
| **LDL-C (mmol/L)** | 2.45 (1.79, 3.27) | 2.48 (1.81, 3.30) | 2.29 (1.59, 3.11) | 0.061 |
| **TG (mmol/L)** | 1.83 (1.21, 2.66) | 1.92 (1.31, 2.84) | 1.34 (0.97, 1.99) | <0.001 |
| **eGFR (mL/min/1.73m2)** | 74.77 (56.80, 89.56) | 77.31 (60.89, 91.37) | 55.59 (41.66, 76.59) | <0.001 |
| **SCr (umol/L)** | 82.00 (66.00, 103.00) | 80.00 (66.00, 98.00) | 94.00 (69.00, 121.75) | <0.001 |
| **UA (umol/L)** | 375.00 (308.00, 452.00) | 378.50 (308.00, 453.25) | 352.00 (299.75, 434.25) | 0.197 |
| **CRP (mg/L)** | 2.33 (0.54, 6.03) | 2.21 (0.52, 5.91) | 2.50 (0.67, 6.74) | 0.174 |
| **WBC (10^9/L)** | 7.05 (5.98, 8.30) | 7.07 (6.10, 8.33) | 6.84 (5.67, 8.18) | 0.154 |
| **HGB (g/L)** | 130.00 (122.00, 140.25) | 133.00 (123.00, 143.00) | 125.00 (111.00, 131.00) | <0.001 |
| **Grip (kg)** | 20.50 (15.85, 27.73) | 21.92 (17.25, 29.79) | 14.60 (10.85, 18.34) | <0.001 |
| **ASM (kg)** | 18.65 (14.92, 22.60) | 19.94 (15.62, 23.23) | 13.54 (12.08, 18.51) | <0.001 |
| **Smoking status (%)** | 146 (17.7) | 132 (19.6) | 14 (9.2) | 0.003 |
| **Alcohol status (%)** | 74 (9.0) | 68 (10.1) | 6 (3.9) | 0.025 |
| **HTN (%)** | 751 (91.1) | 613 (91.2) | 138 (90.8) | 0.991 |
| **DM (%)** | 381 (46.2) | 316 (47.0) | 65 (42.8) | 0.389 |
| **CVD (%)** | 824 (100.0) | 672 (100.0) | 152 (100.0) |  |
| **CKD (%)** | 101 (12.3) | 69 (10.3) | 32 (21.1) | <0.001 |
| **COPD (%)** | 15 (1.8) | 8 (1.2) | 7 (4.6) | 0.012 |
| **CLD (%)** | 4 (0.5) | 3 (0.4) | 1 (0.7) | 1.000 |
| **Arthritis (%)** | 47 (5.7) | 36 (5.4) | 11 (7.2) | 0.478 |

SBP, systolic blood pressure; DBP, diastolic blood pressure; FPG, fasting plasma glucose; HbA1c, hemoglobin A1c; TC, total cholesterol; HDL-C, high-density lipoprotein cholesterol; LDL-C, low-density lipoprotein cholesterol; TG, triglycerides; eGFR, estimated glomerular filtration rate; SCr, serum creatinine; UA, uric acid; CRP, C-reactive protein; WBC, white blood cell count; HGB, hemoglobin; ASM, appendicular skeletal muscle mass; HTN, hypertension; DM, diabetes mellitus; CVD, cardiovascular disease; CKD, chronic kidney disease; COPD, chronic obstructive pulmonary disease; CLD, chronic liver disease.

## 2.4Table S4 Comparison between variables in training set, internal validation set, external validation set and hospital validation set

| **Characteristic** | **Overall** | **Training set** | **Internal validation set** | **External validation set** | **Hospital validation set** | **p-value** |
| --- | --- | --- | --- | --- | --- | --- |
|  | **(n=13834)** | **(n=5082)** | **(n=2178)** | **(n=5750)** | **(n=824)** |  |
| **Age (years)** | 60.00 (53.00, 67.00) | 61.00 (53.00, 67.00) | 61.00 (54.00, 68.00) | 58.00 (51.00, 65.00) | 70.00 (62.00, 78.00) | <0.001 |
| **Gender** |  |  |  |  |  | 0.005 |
| **Male (%)** | 6065 (43.8) | 2251 (44.3) | 943 (43.3) | 2465 (42.9) | 406 (49.3) |  |
| **Female (%)** | 7769 (56.2) | 2831 (55.7) | 1235 (56.7) | 3285 (57.1) | 418 (50.7) |  |
| **Height (cm)** | 157.80 (152.00, 164.30) | 157.40 (151.90, 164.10) | 157.50 (151.60, 163.77) | 157.50 (152.00, 164.00) | 161.00 (156.00, 169.00) | <0.001 |
| **Weight (kg)** | 62.70 (55.90, 70.00) | 62.40 (55.80, 69.80) | 62.50 (55.40, 69.90) | 62.50 (55.90, 69.70) | 66.00 (59.00, 74.43) | <0.001 |
| **SBP (mmHg)** | 130.00 (117.00, 145.00) | 128.50 (116.50, 143.00) | 129.50 (116.00, 144.50) | 130.00 (117.00, 146.00) | 135.00 (123.00, 147.00) | <0.001 |
| **DBP (mmHg)** | 76.50 (68.50, 85.00) | 76.00 (68.50, 84.00) | 76.00 (68.50, 84.50) | 76.50 (69.00, 85.50) | 76.00 (67.00, 85.00) | <0.001 |
| **FPG (mmol/L)** | 5.61 (5.11, 6.38) | 5.41 (5.01, 6.01) | 5.41 (5.01, 6.01) | 5.78 (5.32, 6.46) | 6.97 (5.73, 8.91) | <0.001 |
| **HbA1c (%)** | 5.60 (5.20, 6.10) | 5.90 (5.60, 6.20) | 5.80 (5.50, 6.20) | 5.20 (4.90, 5.50) | 6.10 (5.70, 6.80) | <0.001 |
| **TC (mmol/L)** | 4.83 (4.24, 5.50) | 4.74 (4.18, 5.36) | 4.76 (4.21, 5.41) | 4.98 (4.39, 5.66) | 4.37 (3.58, 5.28) | <0.001 |
| **HDL-C (mmol/L)** | 1.24 (1.05, 1.45) | 1.26 (1.09, 1.45) | 1.26 (1.10, 1.47) | 1.21 (1.00, 1.46) | 1.15 (0.96, 1.35) | <0.001 |
| **LDL-C (mmol/L)** | 2.79 (2.26, 3.35) | 2.64 (2.19, 3.14) | 2.66 (2.16, 3.16) | 3.02 (2.47, 3.64) | 2.45 (1.79, 3.27) | <0.001 |
| **TG (mmol/L)** | 1.40 (0.99, 2.06) | 1.43 (1.02, 2.08) | 1.44 (1.02, 2.08) | 1.32 (0.93, 1.93) | 1.83 (1.21, 2.66) | <0.001 |
| **eGFR (mL/min/1.73m2)** | 103.06 (84.08, 124.12) | 103.74 (84.97, 124.44) | 104.91 (85.66, 126.22) | 105.94 (87.25, 126.61) | 74.77 (56.80, 89.56) | <0.001 |
| **SCr (umol/L)** | 67.80 (57.94, 80.30) | 67.60 (57.90, 80.10) | 66.80 (57.40, 79.50) | 66.93 (57.94, 78.91) | 82.00 (66.00, 103.00) | <0.001 |
| **UA (umol/L)** | 460.00 (378.00, 552.80) | 490.00 (400.00, 590.00) | 480.00 (400.00, 580.00) | 436.80 (362.42, 523.11) | 375.00 (308.00, 452.00) | <0.001 |
| **CRP (mg/L)** | 1.40 (0.74, 2.71) | 1.60 (0.90, 2.80) | 1.60 (0.90, 2.80) | 1.18 (0.63, 2.34) | 2.33 (0.54, 6.03) | <0.001 |
| **WBC (10^9/L)** | 6.00 (5.00, 7.20) | 5.80 (4.86, 6.96) | 5.80 (4.90, 6.90) | 6.02 (5.00, 7.30) | 7.05 (5.98, 8.30) | <0.001 |
| **HGB (g/L)** | 139.00 (128.00, 151.00) | 137.00 (126.00, 149.00) | 136.00 (126.00, 148.00) | 143.00 (131.00, 156.00) | 130.00 (122.00, 140.25) | <0.001 |
| **Grip (kg)** | 28.50 (22.16, 36.20) | 28.15 (22.00, 35.75) | 28.25 (22.25, 35.50) | 30.00 (23.50, 37.95) | 20.50 (15.85, 27.73) | <0.001 |
| **ASM (kg)** | 17.22 (14.47, 21.16) | 17.11 (14.44, 21.07) | 17.14 (14.30, 21.05) | 17.20 (14.52, 21.07) | 18.65 (14.92, 22.60) | <0.001 |
| **Smoking status (%)** | 5166 (37.3) | 2100 (41.3) | 880 (40.4) | 2040 (35.5) | 146 (17.7) | <0.001 |
| **Alcohol status (%)** | 5432 (39.3) | 2290 (45.1) | 951 (43.7) | 2117 (36.8) | 74 (9.0) | <0.001 |
| **HTN (%)** | 6501 (47.0) | 2399 (47.2) | 1013 (46.5) | 2338 (40.7) | 751 (91.1) | <0.001 |
| **DM (%)** | 1938 (14.0) | 708 (13.9) | 308 (14.1) | 541 (9.4) | 381 (46.2) | <0.001 |
| **CVD (%)** | 3941 (28.5) | 1357 (26.7) | 578 (26.5) | 1182 (20.6) | 824 (100.0) | <0.001 |
| **CKD (%)** | 1216 (8.8) | 540 (10.6) | 214 (9.8) | 361 (6.3) | 101 (12.3) | <0.001 |
| **COPD (%)** | 1568 (11.3) | 693 (13.6) | 329 (15.1) | 531 (9.2) | 15 (1.8) | <0.001 |
| **CLD (%)** | 711 (5.1) | 348 (6.8) | 147 (6.7) | 212 (3.7) | 4 (0.5) | <0.001 |
| **Arthritis (%)** | 5367 (38.8) | 2279 (44.8) | 957 (43.9) | 2084 (36.2) | 47 (5.7) | <0.001 |

SBP, systolic blood pressure; DBP, diastolic blood pressure; FPG, fasting plasma glucose; HbA1c, hemoglobin A1c; TC, total cholesterol; HDL-C, high-density lipoprotein cholesterol; LDL-C, low-density lipoprotein cholesterol; TG, triglycerides; eGFR, estimated glomerular filtration rate; SCr, serum creatinine; UA, uric acid; CRP, C-reactive protein; WBC, white blood cell count; HGB, hemoglobin; ASM, appendicular skeletal muscle mass; HTN, hypertension; DM, diabetes mellitus; CVD, cardiovascular disease; CKD, chronic kidney disease; COPD, chronic obstructive pulmonary disease; CLD, chronic liver disease.

## 2.4Table S5 Multivariable logistic regression coefficients and odds ratios for the sarcopenia prediction model in CKM patients.

| **Variables** | **β** | **OR** | **95% CI** | **P** |
| --- | --- | --- | --- | --- |
| **Intercept** | -6.437 | - | - | - |
| **Age** | 0.103 | 1.108 | (1.095, 1.122) | <0.001 |
| **Smoking status** | 0.581 | 1.789 | (1.404, 2.278) | <0.001 |
| **HDL-C** | 0.936 | 2.55 | (1.773, 3.667) | <0.001 |
| **TG** | -0.269 | 0.764 | (0.658, 0.888) | <0.001 |
| **UA (per 100 µmol/L)** | -0.014 | 0.986 | (0.979, 0.994) | 0.001 |
| **CRP** | 0.021 | 1.021 | (1.010, 1.033) | <0.001 |
| **HGB** | -0.024 | 0.976 | (0.970, 0.983) | <0.001 |
| **COPD** | 0.456 | 1.578 | (1.210, 2.057) | 0.001 |
| **CLD** | 0.539 | 1.714 | (1.184, 2.482) | 0.004 |

HDL-C, High-Density Lipoprotein Cholesterol; TG, Triglycerides; UA, Uric Acid; CRP, C-reactive protein; HGB, Hemoglobin; COPD, Chronic obstructive pulmonary disease; CLD, Chronic Liver Disease
